# The Partial Iliopubic Tract Resection Technique for Incarcerated Femoral Hernia: A Case Series and a Literature Review

**DOI:** 10.7759/cureus.62985

**Published:** 2024-06-23

**Authors:** Kioto Yokoyama, Keisuke Tomoda, Satoru Takayama

**Affiliations:** 1 General Surgery, Nagoya Tokushukai General Hospital, Kasugai, JPN

**Keywords:** incarcerated femoral hernia, laparoscopic repair, iliopubic tract division, bowel obstruction, minimally invasive surgery

## Abstract

Femoral hernias have a high incarceration rate, often necessitating urgent surgical intervention. In this report, we present a safe and reproducible laparoscopic technique for incarcerated femoral hernias with bowel involvement, including repair.

Between December 2022 and May 2023, three female patients with incarcerated femoral hernias underwent urgent laparoscopic surgery. All patients presented with abdominal pain and were diagnosed with small bowel incarceration using computed tomography. Under laparoscopy, we confirmed intestinal incarceration and performed a standard transabdominal preperitoneal approach to identify the hernia defects. The iliopubic tract on the abdominal side of the hernia defect was carefully dissected using an energy device to enlarge the hernia orifice. A spontaneous reduction of the incarcerated intestine was achieved. After confirming the absence of bowel perforation, mesh was placed to repair the hernia. Following peritoneal closure, the affected part of the intestine was extracorporeally resected and anastomosed. We performed this technique on three patients, all of whom were later discharged without complications.

In conclusion, for incarcerated femoral hernias with bowel obstruction, laparoscopic partial division of the iliopubic tract enables an easy, safe, and reproducible approach to incarceration release and subsequent hernia repair.

## Introduction

Femoral hernias, although less common than inguinal hernias, pose a significantly high risk of incarceration, with 35-43% of cases requiring emergency surgery, often involving bowel resection [1,2]. Among older adults, reports suggest a mortality rate of approximately 5% in patients who undergo emergency surgery for femoral hernia [3-5]. This underscores the critical importance of prompt and effective treatment for femoral hernia incarceration within the context of acute abdominal conditions. Recently, emergency laparoscopic surgery has gained traction and its effectiveness has been well substantiated. Femoral hernias are prone to incarceration owing to the strong ligamentous structure of the femoral ring.

We present a series of three consecutive cases of incarcerated femoral hernias treated with emergency laparoscopic surgery. Our approach involved partial resection of the iliopubic tract (IPT) and enlargement of the hernia orifice, enabling a safe release of the incarcerated content. Concurrent hernia repair was achieved using the same laparoscopic technique. This minimally invasive approach is considered safe and reproducible, and does not require advanced technical skills. Here, we discussed our experience with emergency surgery for incarcerated femoral hernias and described the surgical technique based on a review of the literature.

## Case presentation

Between December 2022 and May 2023, three female patients with incarcerated right femoral hernias were admitted as emergency cases. All patients presented with abdominal pain and were transported to the hospital via emergency medical services. A reduction in hernia content was not possible in all instances. Preoperative computed tomography (CT) scans confirmed small bowel incarceration with intestinal obstruction, prompting urgent laparoscopic surgery.

**Figure 1:**
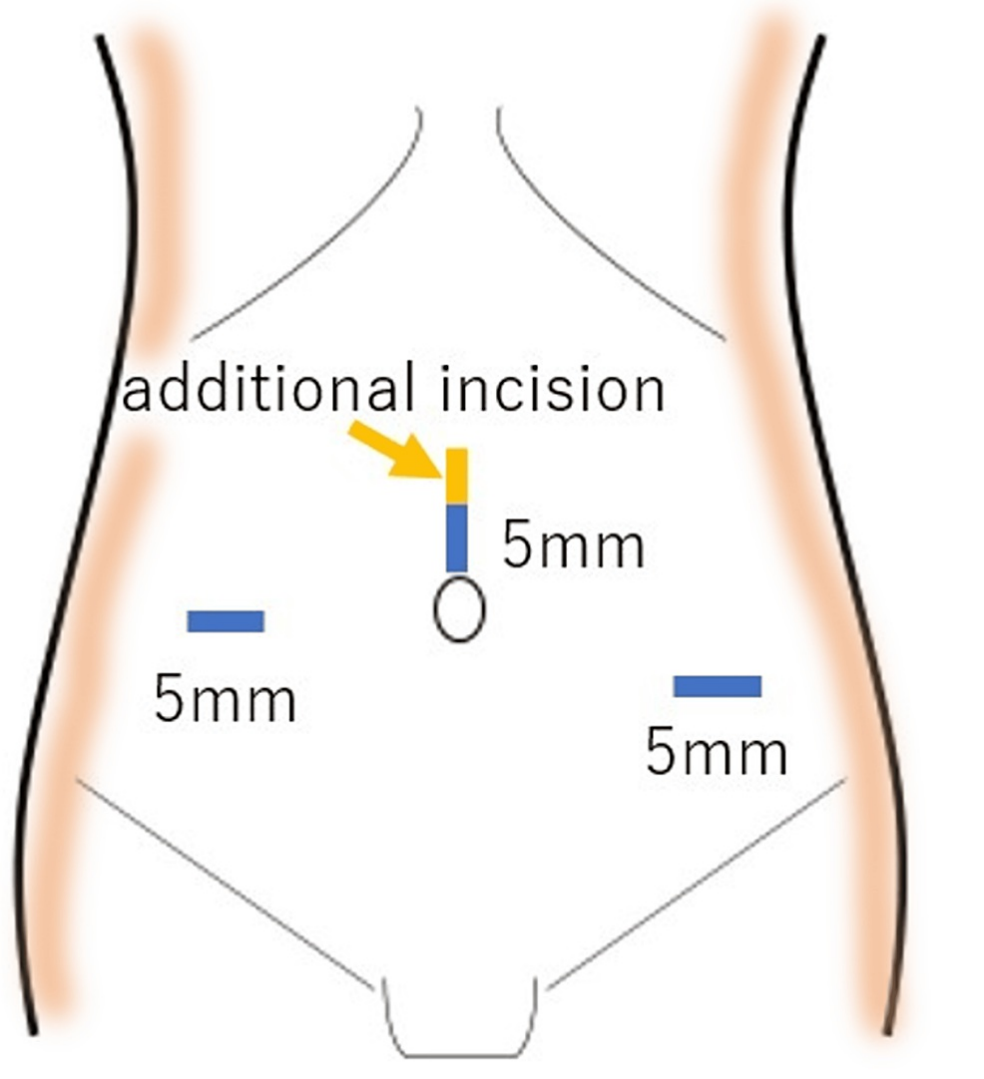
Port placement and skin incision Three 5 mm ports were placed as shown in this figure. Additionally, a midline incision was made after hernia repair to perform bowel resection. Image created by the authors.

All patients underwent laparoscopic repair of a right femoral hernia with intestinal obstruction using general anesthesia. A vertical incision was made above the umbilicus and a 5-mm trocar was inserted using the optical method with a 5-mm direct-view endoscope. The abdominal cavity was then examined to assess for adhesions. Further, a 5-mm port was placed >4 fingerbreadths outward to the right from the first port. Additionally, another 5 mm port was inserted between the first port and the left anterior superior iliac spine, positioned >4 fingerbreadths away from the first port (Figure 1). Gauze manipulation was performed using 3 mm laparoscopic grasper forceps.

**Figure 2:**
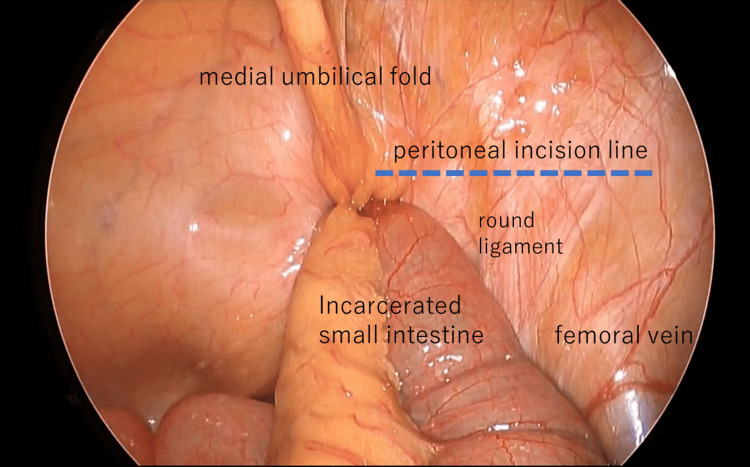
Right femoral hernia with incarcerated small intestine

**Figure 3:**
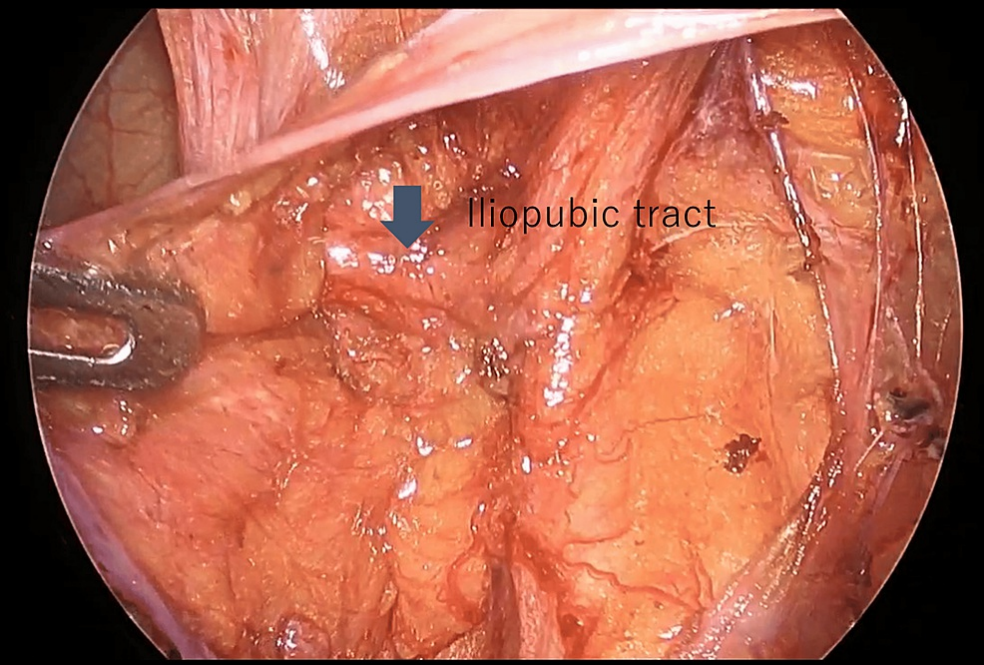
IPT findings on laparoscopy After dissecting the peritoneum and separating the preperitoneal fat, the IPT that forms the head side of the hernia gate is identified. IPT: iliopubic tract

**Figure 4:**
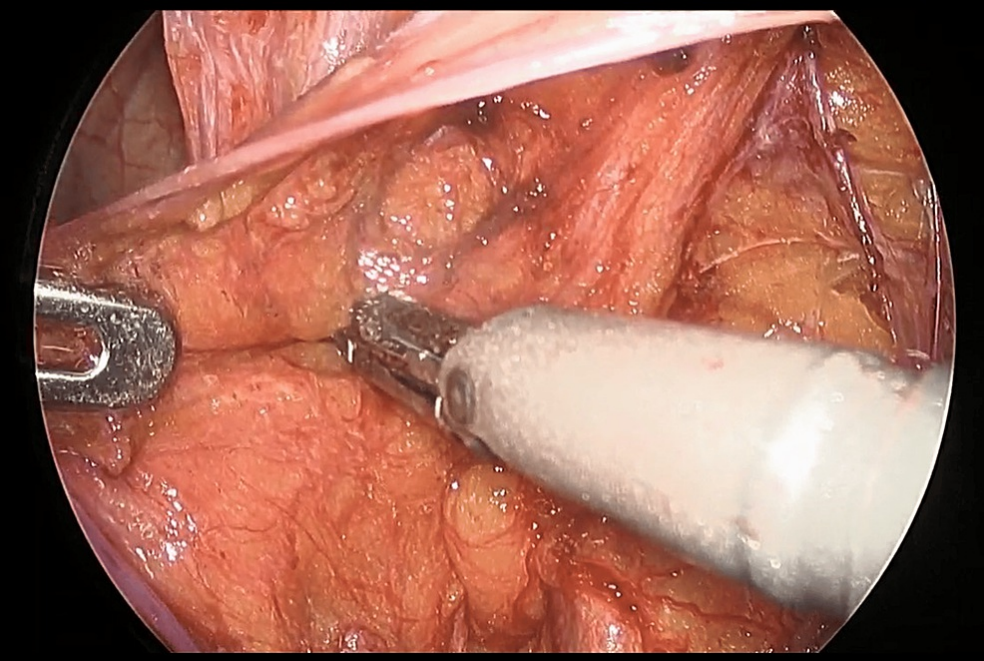
Partial iliopubic tract resection Positioning the tissue padding of the LSC with the bowel side up, taking great care to avoid intestinal injury, and then detaching the IPT. LCS: laparoscopic coagulation shears; IPT: iliopubic tract

**Figure 5:**
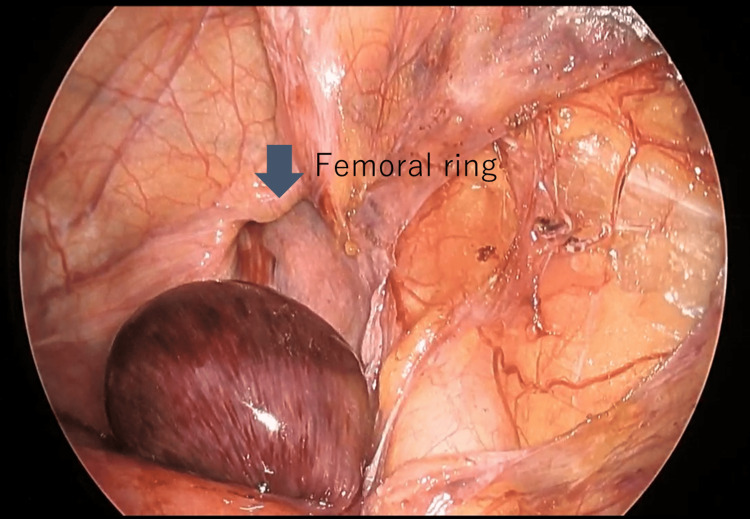
The reduction of hernia incarceration By detaching the IPT, it is possible to release the hernia without the need for intestinal traction. IPT: iliopubic tract

In all three cases, the small intestine was incarcerated in the femoral ring. Intestinal traction was minimized owing to the risk of perforation. The initial step involved creating a peritoneal incision extending from the hernia orifice to the medial umbilical fold (Figure 2). The preperitoneal space was then dissected and the round ligament of the uterus was separated using laparoscopic coagulation shears (LCS). The preperitoneal space was expanded by dissecting the hernial orifice, allowing IPT identification (Figure 3). Subsequently, the space between the IPT and incarcerated intestine was gently dissected using forceps to create a slight separation, followed by partial division of the IPT using LCS (Figure 4). During this process, the tissue padding of the LCS was oriented toward the intestinal side to ensure adequate precautions against intestinal injury. The incarcerated small intestine was naturally released by dissecting a few millimeters of the IPT (Figure 5). None of the three patients exhibited perforation of the small intestine.

**Figure 6:**
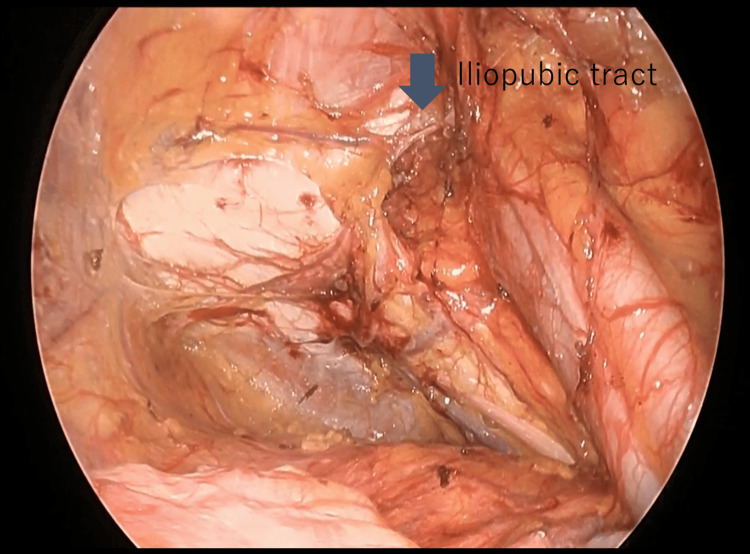
A complete dissection of the area surrounding the hernia orifice

**Figure 7:**
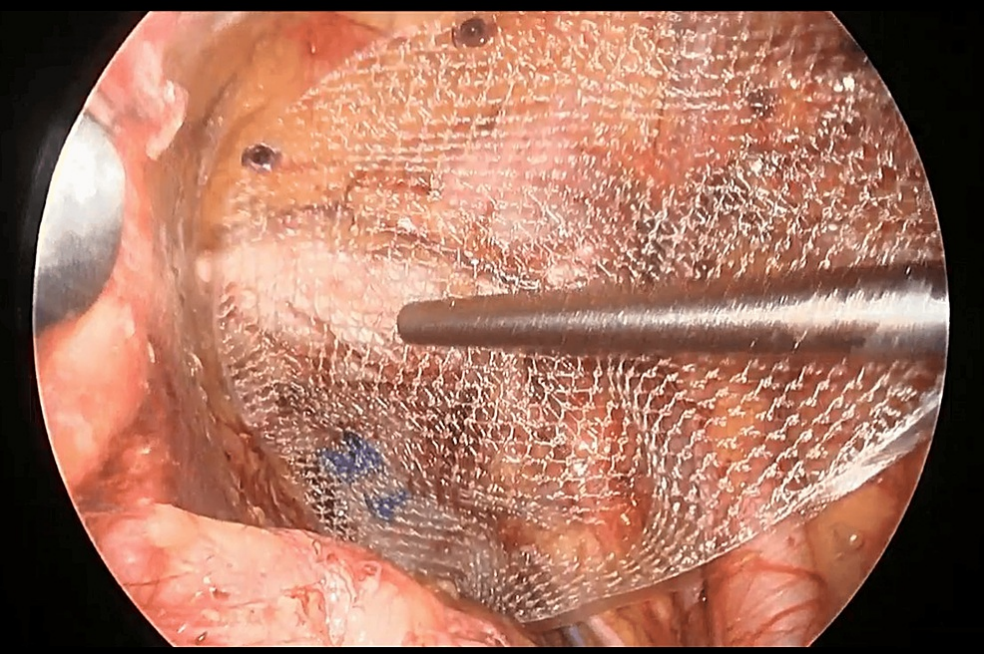
Place a mesh and secure it with tacks

**Figure 8:**
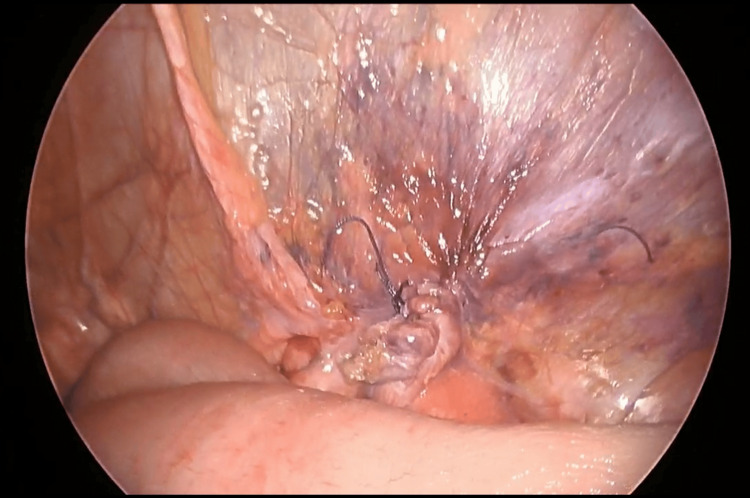
Closure of the peritoneum The peritoneum was closed using a 3-0 braided absorbable suture.

Dissection around the hernia orifice was adequately performed, and a lightweight monofilament polypropylene mesh (size: 7.9 cm × 13.4 cm in two cases, 10.3 cm × 15.7 cm in one case) was placed for repair (Figures 6, 7). Absorbable tacks (five per patient) were used to secure the mesh to Cooper's ligament, inferior epigastric vessels on both sides, rectus abdominis, and external oblique aponeurosis. Furthermore, the peritoneum was closed using a 3-0 braided absorbable suture (Figure 8).

In all cases, the reduced incarcerated small intestine was necrotic with no prospect of blood flow recovery. The incision at the umbilicus was extended by 3 cm and the wound edges were protected. The necrotic small intestine was then exteriorized, resected, and anastomosed.

The median operative time was 135 minutes (range: 103-233 minutes). One patient had extensive adhesions in the right lower abdomen, which led to a longer surgical duration. All patients experienced positive postoperative recovery and were discharged within a week. One patient developed a postoperative seroma that resolved with observation. The mean follow-up period was three months (range: 1-6 months), with no hernia recurrences reported (Table 1).

**Table 1:** Clinical and operative characteristics of patients operated for incarcerated femoral hernias BMI: body mass index; M: 7.9 cm × 13.4 cm; L: 10.3 cm × 15.7 cm

Age	Sex	Height/BMI	Symptoms	Duration of Symptoms (h)	Operating Time (min)	Mesh Size	Postop Stay (day)	Complications	Recurrence
70	F	152/15.6	Abdominal pain	3	103	M	5	Seroma	No
79	F	156/16.8	Abdominal pain	3	135	M	7	None	No
96	F	138/24.2	Abdominal pain	7	233	L	5	None	No

## Discussion

**Figure 9:**
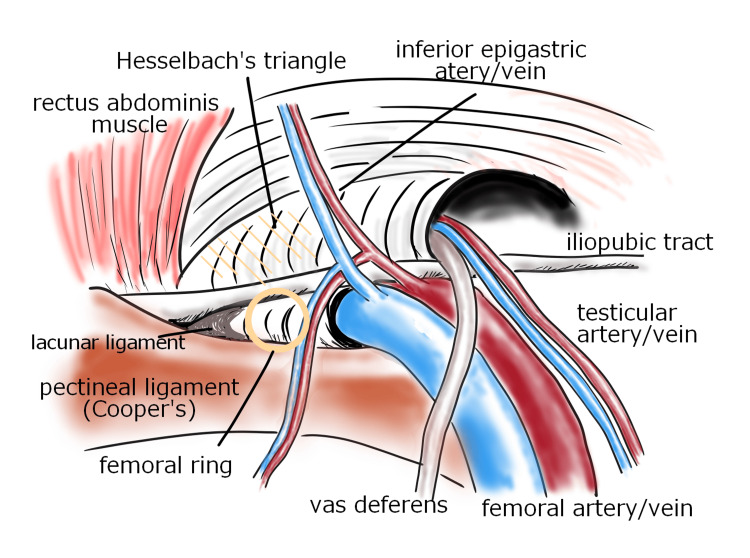
Anatomy around the femoral ring Image created by the authors.

Femoral hernias have a high risk of incarceration because of the robust ligamentous framework of the femoral ring. Notably, the outer aspect is formed by the lacunar ligament, the superior aspect by the IPT, the inferior aspect by the pectinal ligament, and the medial aspect by the vascular sheath (Figure 9).

The management of incarcerated femoral hernias poses specific challenges and risks, necessitating careful consideration of surgical approaches. This report highlights the efficacy of a laparoscopic technique for releasing incarcerated femoral hernias with bowel involvement and subsequent repair.

Various surgical approaches have been employed for emergency femoral hernia repair, with the anterior approach (open surgery) commonly utilized. However, the effectiveness of the transabdominal preperitoneal (TAPP) approach has been widely reported recently.

The introduction of laparoscopic techniques in 1993 marked a turning point in incarcerated hernia treatment [6]. The European Association for Endoscopic Surgery's 2013 endorsement of laparoscopy for incarcerated hernias, advocating for hernia reduction under laparoscopic guidance, further solidified its role [7].

Recent reports have substantiated the advantages of laparoscopic approaches, such as decreased laparotomy rates, reduced wound infections, and shorter hospital stays, compared with open approaches. TAPP repair demonstrates comparable recurrence rates, along with reduced postoperative discomfort and a quicker return to normalcy, solidifying its efficacy over the anterior approach [8-10]. Notably, the emergency TAPP procedure allows for crucial intraoperative bowel assessment and confirmation of bilateral lesions. Careful laparoscopic evaluation can reveal recovery of normal coloration in the incarcerated bowels, contributing to the avoidance of unnecessary bowel resections [11-13].

In our study, all patients presented with challenging manual reductions, prompting TAPP procedures to release the hernia without the need for intestinal traction. Although all cases exhibited bowel necrosis, our technique prevented further injury or perforation, enabling bowel resection after hernia repair.

Our procedures were conducted by surgeons in their fourth to fifth postgraduate years under the guidance of experienced hernia surgeons. This suggests that the technique can be safely performed by less experienced surgeons under appropriate supervision and shared procedural understanding. The technique’s high reproducibility was evident in its successful application across all cases without significant variations.

Enlarging the hernial defect during repair may appear counterintuitive; however, the primary aim is to reduce the risk of intestinal perforation. Hence, enlargement of the hernial defect is justified because sufficient mesh coverage can be achieved with accurate placement.

The discussion of mesh infection is particularly relevant in emergency hernia repair, as mesh implantation is considered a potential risk factor for infection development [14,15]. Mesh infection is a serious complication that often necessitates mesh removal in a secondary procedure, imposing a significant burden on patients [16]. However, mesh placement allows for a single-stage surgery, reducing the length of hospital stay. Given these considerations, the decision to use meshes requires careful consideration.

Recent reports suggest that mesh repair in acute hernias may decrease recurrence risk [17,18]. The primary risk factor for mesh infection is associated with the mesh’s filament type and pore size. Multifilament meshes are more susceptible to infection than monofilament meshes. Furthermore, lightweight meshes with larger pores offer advantages, such as increased elasticity, greater flexibility, and reduced patient discomfort. Additionally, larger pores are associated with decreased inflammation and reduced risk of infection. Therefore, selecting a lightweight large-pore monofilament mesh is recommended. In our cases, all patients received repairs using a lightweight monofilament polypropylene mesh with large pores [19].

Prophylactic antibiotic use is recommended, and peritoneal irrigation following hernia reduction aligns with the practices of other authors [20].

## Conclusions

Emergency laparoscopic surgery is beneficial for the treatment of incarcerated femoral hernias. Detachment of the IPT with concomitant enlargement of the hernia orifice minimizes the risk of intestinal injury during incarceration release, facilitating successful repair. This minimally invasive technique offers potential benefits for patient recovery.
